# Biochemical Monitoring of Spinal Cord Injury by FT-IR Spectroscopy—Effects of Therapeutic Alginate Implant in Rat Models

**DOI:** 10.1371/journal.pone.0142660

**Published:** 2015-11-11

**Authors:** Sandra Tamosaityte, Roberta Galli, Ortrud Uckermann, Kerim H. Sitoci-Ficici, Robert Later, Rudolf Beiermeister, Falko Doberenz, Michael Gelinsky, Elke Leipnitz, Gabriele Schackert, Edmund Koch, Valdas Sablinskas, Gerald Steiner, Matthias Kirsch

**Affiliations:** 1 Clinical Sensoring and Monitoring, Department of Anesthesiology and Intensive Care Medicine, Faculty of Medicine, TU Dresden, Dresden, Germany; 2 Faculty of Physics, dept. of General Physics and Spectroscopy, Vilnius University, Vilnius, Lithuania; 3 Neurosurgery, Carl Gustav Carus University Hospital, TU Dresden, Dresden, Germany; 4 Centre for Translational Bone, Joint and Soft Tissue Research, TU Dresden, Dresden, Germany; 5 CRTD / DFG-Center for Regenerative Therapies Dresden—Cluster of Excellence, TU Dresden, Dresden, Germany; The University of Akron, UNITED STATES

## Abstract

Spinal cord injury (SCI) induces complex biochemical changes, which result in inhibition of nervous tissue regeneration abilities. In this study, Fourier-transform infrared (FT-IR) spectroscopy was applied to assess the outcomes of implants made of a novel type of non-functionalized soft calcium alginate hydrogel in a rat model of spinal cord hemisection (n = 28). Using FT-IR spectroscopic imaging, we evaluated the stability of the implants and the effects on morphology and biochemistry of the injured tissue one and six months after injury. A semi-quantitative evaluation of the distribution of lipids and collagen showed that alginate significantly reduced injury-induced demyelination of the contralateral white matter and fibrotic scarring in the chronic state after SCI. The spectral information enabled to detect and localize the alginate hydrogel at the lesion site and proved its long-term persistence in vivo. These findings demonstrate a positive impact of alginate hydrogel on recovery after SCI and prove FT-IR spectroscopic imaging as alternative method to evaluate and optimize future SCI repair strategies.

## Introduction

In traumatic spinal cord injury (SCI) necrotic cell death and vascular damage take place at the lesion site immediately after the trauma. A cascade of secondary events including inflammation, edema and ischemia leads to additional cell death, demyelination and axonal degeneration, which culminate in further loss of nervous functions [1]. This cascade leads to the formation of a scar composed by reactive astrocytes, proteoglycans and extracellular matrix. Especially in case of lacerating injury, a dense fibrous scar surrounded by a glial scar is observed [2].

Spinal cord tissue of mammals has a limited ability to regenerate and it is well known that scarring creates an inhibitory environment for axonal regeneration [3]. However, it was already demonstrated that also axons of the central nervous system (CNS) hold the potential to regrow if an appropriate milieu is provided [4]. The creation of a permissive and promoting environment that allows axonal regrowth across the lesion site in the CNS is in the focus of the research for new therapies [5].

Several studies investigated interventions that include the application of growth and neurotropic factors [6–9], as well as neuroprotective and neutralizing drugs [10–13]. Hydrogels are used as permissive space-filling agents that can be functionalized for delivery of neuroprotective or neurotrophic factors at the lesion site [14]. Hydrogel structure can be modified not only to attach therapeutic molecules, but also to tailor stiffness, permeability, swelling, strength, degradation rate and porosity to match axons’ guidance requirements [15–17].

Alginate is a biopolymer extracted from brown algae that forms hydrogels in aqueous solutions [18]. Since the first trials over a decade ago, implants of alginate hydrogels functionalized with signaling molecules were found to enhance elongation of regenerating axons [19,20]. While different research groups focused on modification of alginate hydrogel to form composite structures [21,22] and incorporate neurotrophic [23–25] or neuroprotective agents [26–28], recent studies demonstrated that soft alginate hydrogels formed by crosslinking with Ca^2+^, Ba^2+^ or Sr^2+^ are intrinsically neurotrophic and neuroprotective, by supporting neurite growth in vitro and protecting neuronal cells against oxidative stress without need of any functionalization [29]. Furthermore, soft Ca^2+^-alginate hydrogels maintain their mass and volume after immersion in saline solution, which makes them interesting for in vivo applications [30].

Vibrational spectroscopic techniques represent an alternative approach for label-free and non-destructive biochemical tissue characterization and provide the tissue’s molecular composition [31–33]. They enable the analysis and classification of pathophysiological tissue alterations [34] and can be transferred into an in vivo setting [35]. We already reported that infrared micro-spectroscopy is a promising tool for mapping the different degenerative areas of SCI in a rat model [36], enabling to retrieve demyelination, inflammation and collagenous scarring.

In this study Fourier-transform infrared (FT-IR) spectroscopic imaging was employed to obtain quantitative information on the therapeutic impact of a nonfunctionalized soft Ca^2+^-alginate hydrogel implant in a rat model of SCI. We used FT-IR spectroscopy to characterize the implant, evaluate possible side effects and monitor the effect of the therapeutic intervention on CNS tissue degeneration and repair. In order to address sub-acute and long term effects of the implants, animals were investigated one and six months after injury.

## Materials and Methods

### Alginate hydrogel implants

Ultrapure low viscosity sodium alginate (Pronova up lvm, Novamatrix, Sandvika, Norway) with a ratio of guluronic acid to mannuronic acid building blocks of 1 was used. A 4% alginate solution (sol) in deion. water was prepared by stirring for 12 h and sterile filtrated with a 0.45 μm filter. 700 μl of this sol were placed in a petri dish (ø 6 cm), incubated at room temperature for 20 min, overlaid with 10 ml crosslinking solution containing 4 mM CaCl_2_ and 150 mM NaCl and incubated at room temperature for 12 h. Afterwards, the crosslinking solution was removed and the samples were overlaid with 10 ml of a solution containing 2 mM CaCl_2_ and 150 mM NaCl until utilization. The hydrogel was manually cut in blocks with dimension 2 mm × 2 mm × 1.5 mm to prepare the implants (S1 Fig).

### Rheological characterization

The rheological characterization of the alginate hydrogel was carried out on a Rheotest RN 4.1 instrument (Rheotest Medingen GmbH, Medingen, Germany). A measurement cell with parallel plate geometry (P1) was used. A stress sweep was performed under a constant frequency of 1 Hz at a temperature of 37°C to obtain the storage modulus. The shear stress and deformation were recorded while the amplitude was linear increased from 1 to 100 Pa.

### Ethics statement

All animal experiments were performed in accordance with the guidelines of the Dresden University of Technology, based on national laws that are in full agreement with the European Union directive on animal experimentation. They were approved by the Dresden Regional Council (Regierungspräsidium Dresden), Germany (AZ 24–9168.11-1/2013-37). All efforts were made to minimize animal suffering. Surgery was performed on rats under anesthesia with an intraperitoneal injection of xylazine / ketamine (10 mg / kg xylazine; 90 mg / kg ketamine). In the first two postoperative days, analgesia was achieved with carprofen (5 mg / kg) once a day subcutaneously. Rarely, buprenorphine (0.05 mg / kg) was needed for an appropriate analgesia. The bladder was expressed by gentle massage twice daily during the first two weeks after surgery until the spontaneous urination or reflex bladder emerged.

### Animal experiments

28 female Wistar rats, aged 16 weeks and weighting between 200 g and 250 g, were used. They were anesthetized and a hemisection at the level T9 of the thoracic vertebrae of the spinal cord was surgically induced under a surgical microscope. The left side of the spinal cord was bisected along a medial longitudinal plane for a length of 2 mm as described earlier [37]. 14 randomly selected rats received alginate hydrogel into the lesion. The blocks of alginate hydrogel were transferred to the animal using a surgical micro-spoon spatula and delivered in the hemisection. No therapies were attempted for the remaining 14 rats (control). The musculature and the thin superficial muscle layer were closed and animals were allowed to recover on a commercial electric heating pad.

Seven rats of the control group and seven rats with alginate implant were perfusion-fixed using 4% (w/v) paraformaldehyde in tris-buffered saline at one and six months after surgery. The spinal cords were isolated and cryoprotected in rising sucrose concentration (10% (w/v) for 24 h and 30% (w/v) for 24 h), embedded in tissue freezing medium (Leica, Nussloch, Germany) and frozen on dry ice. Finally, 16 μm thick longitudinal cryosections were prepared on CaF_2_ slides or on glass slides. The cryosections were 20 mm long and the lesion was located roughly in the center. Cryosections were stored at − 20°C until use.

### FT–IR spectroscopic data acquisition

Infrared spectra were acquired in transmission mode with a FT-IR spectrometer Tensor 27 equipped with an infrared microscope Hyperion 3000 (both from Bruker Optic GmbH, Ettlingen, Germany). A 15× Cassegrain objective (0.4 NA) imaged an area of 175 μm × 175 μm. The radiation was collected by a 64 × 64 Mercury Cadmium Telluride (MCT) focal plane array detector. 8 × 8 binning was applied and the spectral resolution was set to 6 cm^-1^. A background spectrum was recorded on a clean position of the CaF_2_ slide. Composite images of several square millimeters depending on the sample size (max. 116 × 18 fields of view, 20.4 mm × 3.2 mm) were captured in an automatized step-wise manner by moving the sample stage. For each pixel, 8 interferograms were collected, co-added and Fourier transformed by applying Blackman–Harris apodization and zero filling factor of 0. Each spectrum was ratioed to the background spectrum and the transmission spectra were converted to absorbance values.

### Reference spectra of pure materials

The reference alginate hydrogel sample was prepared by embedding, freezing and sectioning; cryosections 16 μm thick were placed on a CaF_2_ slide. The reference spectrum of collagen was acquired from collagen IV from human placenta (Sigma−Aldrich, Steinheim, Germany). The reference spectrum of sucrose was acquired from sucrose solution dried on a CaF_2_ slide. Reference spectra were collected in transmission mode with a single-channel MCT detector using the same acquisition parameters as for imaging of spinal cord samples.

### Data processing and analysis

An atmospheric compensation was calculated to subtract contributions of residual water vapor bands from the spectra. FT-IR data was then reduced to the fingerprint region (900–1800 cm^-1^) and baseline corrected in OPUS 7.2 (Bruker Optic GmbH, Ettlingen, Germany).

Further processing and analysis were performed using Matlab Packages (version 7, Math Works Inc. Natick, MA, USA). The integral intensity of the amide I band was calculated in the spectral range 1633–1673 cm^-1^ and used as a marker to identify the tissue. Spectra of embedding medium and of the CaF_2_ slide displaying absorbance value of amide I < 0.3 were excluded. The selected spectra were vector normalized.

A semi-quantification of lipid content was based on the integral intensities calculated in the following spectral ranges: 1218–1232 cm^-1^, 1459–1473 cm^-1^ and 1728–1742 cm^-1^. Regions of interest (ROIs) of 0.2 cm^2^ were chosen contralateral to the lesion. Mean band intensities were normalized to the intensities calculated the same white matter tract caudal to the lesion (4.5 mm away from the hemisection center).

To assess the extension of the scar, the intensity values of the spectral band at 1242 cm^-1^ were calculated along a line crossing the scar center. Pixels with intensity values above the background of the nervous tissue were assigned to fibrous tissue and used to evaluate the thickness of the scar (S2 Fig).

Data is expressed as mean ± SEM. For comparison of control and alginate group, a two-tailed t-test was performed using Graph Pad Prism 6.0 (Graph Pad Software Inc., La Jolla, CA, USA).

### Stainings

Consecutive sections of spinal cords were fixed in methanol-acetone (1:1) and stained with hematoxylin and eosin (H&E) in order to provide a reference for spectroscopic imaging. Sections were washed in distilled water and incubated in Meyer’s hematoxylin/hemalum for 3 min. After washing in distilled water, sections were briefly destained in HCl-ethanol and washed using tap water for 5 min. After 3 min of staining in eosin (1% (w/v) eosin G in 80% ethanol), the sections were dehydrated with rising ethanol concentrations, cleared in xylene and coverslipped using DePeX mounting medium (SERVA Electrophoresis GmbH, Heidelberg, Germany).

To provide a reference for spectroscopic imaging of alginate hydrogel implants in spinal cord tissue, sections were stained with alcian blue which is specific for polysaccharides. Alcian blue solution (pH = 2) was placed on sections and the staining success was observed under the microscope. Then, the sections were washed with running tap water for 3 min and stained with nuclear fast red for 10 min. After 10 min of washing with running tap water, the sections were dehydrated with rising ethanol concentrations, cleared in xylene and coverslipped using DePeX mounting medium.

## Results and Discussion

**Fig 1:**
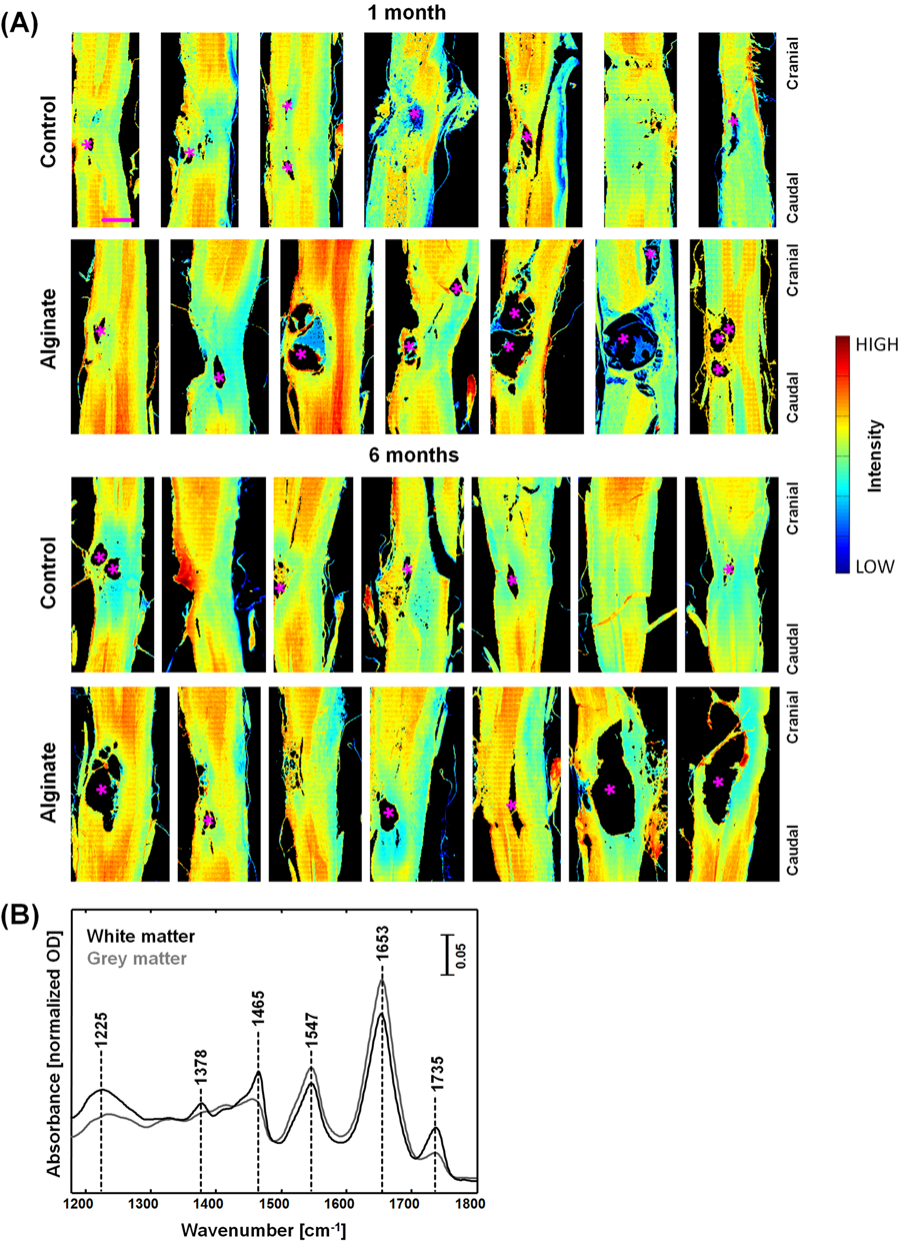
IR spectroscopic imaging of SCI in rat models with and without alginate hydrogel implant at one and six months after injury. (A) Spectroscopic images of longitudinal cryosections of the investigated samples, showing the intensity of the amide I band at 1653 cm^-1^; asterisks (*) indicate large cysts. Scale bar: 1 mm. (B) Representative IR spectra of white and grey matter.

IR imaging was performed on longitudinal sections of rat spinal cords (n = 28) harvested one month and six months after injury. The IR spectroscopic images in Fig 1A show the intensity of the amide I band at 1653 cm^-1^: They represent the distribution of proteins and reveal the overall structure of the samples. Different regions were identified: White matter tracts are characterized by a lower amide I intensity (light blue and green pixels) while grey matter displays a higher amide I intensity (yellow and red pixels). At the site of the lesion the regular structure of alternating strands of gray and white matter is disrupted. The lesion site is located approximately in the center of the samples shown in the figure. Cysts with dimension up to approximately 2 mm were observed anterior and/or posterior to the lesion core (asterisks in Fig 1A). Images of H&E stained consecutive sections are shown for reference in S3 Fig.

Fig 1B shows representative IR spectra of white and grey matter in the rat spinal cord. The spectral region 900^–^1180 cm^-1^ was excluded from all analyses as it is dominated by contribution of the absorption of sucrose (S4 Fig), which was used during the preparation of samples for cryoprotection in order to preserve tissue structure and morphology.

The most prominent bands of spinal cord tissue are amide I at 1653 cm^-1^ and amide II at 1547 cm^-1^, which are characteristic vibration modes of proteins and polypeptides. The bands at 1735 cm^-1^ is assigned to ν_s_(C = O), the band at 1466 cm^-1^ to δ[(CH_2_)], the band at 1378 cm^-1^ to δ[(CH_3_)] and the band at 1225 cm^-1^ to ν_as_(PO_2_^−^) [36,38]. In the CNS nervous tissue, they mainly account for molecular vibrations of lipids and phospholipids. These lipid-related bands are more intense in white matter, which is mainly composed of myelinated axons, which are rich in cholesterol and phospholipids [39]. In contrast, grey matter mostly consists of neuronal cell bodies and contains only a few myelinated axons.

**Fig 2:**
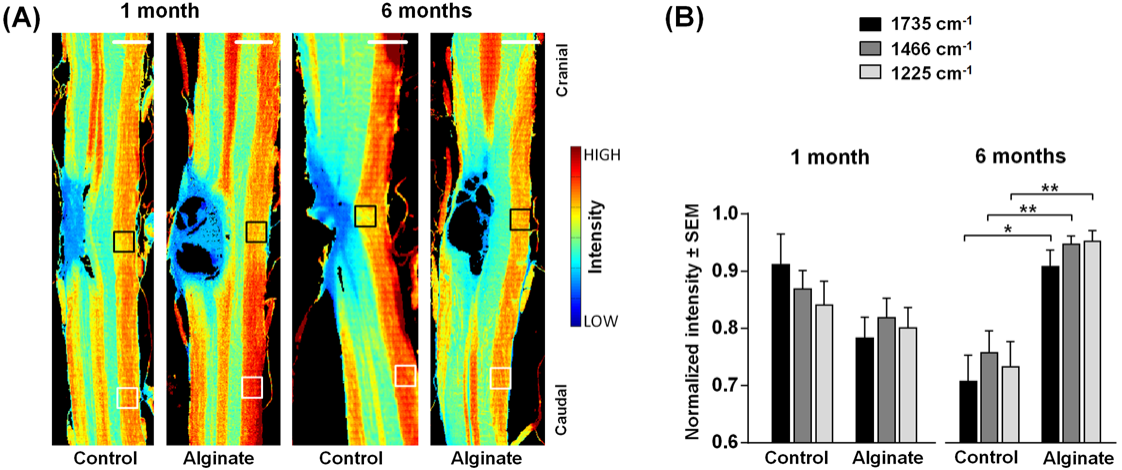
Analysis of the contralateral nervous tissue. (A) Spectroscopic images showing the intensity of the band at 1735 cm^-1^ (ν_s_(C = O)). They depict the distribution of lipids in control and alginate-implanted samples one and six months post-injury. Scale bar: 1 mm. (B) Intensities of the bands at 1735 cm^-1^, 1466 cm^-1^ (δ[(CH_2_)], also showing the distribution of lipids) and 1225 cm^-1^ (ν_as_(PO_2_^−^), showing the distribution of phospholipids) in the white matter contralateral to the lesion indicated by the black boxes in panel A normalized for each samples to the intensity in the region indicated by white boxes in panel A. For each group n = 5–6. Two-tailed t-test, *: p < 0.05, **: p < 0.01.

Following SCI, the alteration of lipid content is a hallmark of severity of the axonal degeneration and, on the other hand, of the potential of axons to regenerate [40]. Following the initial decrease of total lipid content due to damage of neuronal membranes and ischemia-induced lipid peroxidation [41], the demyelination of the nervous tissue adjacent to the site of the injury proceeds in the secondary phase of SCI. Therefore, the distribution of lipids was investigated by analyzing the spectral bands that are attributed to lipids, in order to investigate the effect of the soft Ca^2+^-alginate hydrogel implant. Representative examples of spectral images obtained by plotting the intensity of the band at 1735 cm^-1^ are shown in Fig 2A for each experimental group; images of all samples are shown in S5 Fig. They visualize the spatial distribution of lipids within the tissue sections. White matter (red/yellow pixels) and gray matter (green pixels) are readily localized based on their different lipid content, while the lesion center is strongly depleted of lipids (blue pixels).

Furthermore, a reduced lipid content that represents injury-induced demyelination was observed in the white matter contralateral to the lesion in the control samples. The respective intensities in the regions indicated by the black boxes in Fig 2A were lower than the intensities of the same white matter tract distant to the lesion (positions indicated by white boxes). In the alginate sample harvested six months after injury this contralateral demyelination is not evident.

The intensity of the band at 1735 cm^-1^ and of two other bands that correspond to lipids, e. g. at 1225 cm^-1^ and at 1466 cm^-1^, was analyzed one month and six months after SCI (Fig 2B) to further evaluate the impact of the alginate implant on injury-induced demyelination of the contralateral white matter tract. A reduction of lipid-related bands was observed in all experimental groups, but with a different extent. One month post-injury no significant difference between the control and the alginate group was found. After six months, significantly higher intensities indicating higher lipid content were observed in the samples with alginate (Fig 2B, two-tailed t-test, p = 0.019, p = 0.0095, p = 0.0095 for 1735 cm^-1^, 1466 cm^-1^ and 1225 cm^-1^ bands, respectively).

Degradation of contralateral white matter takes place in rat models at the chronic stage after hemisection, it includes demyelination and affects function. [42,43] Our findings suggest that the non-functionalized Ca^2+^-alginate hydrogel has a positive impact on presence of myelin in contralateral white matter. This might result from either reduced demyelination in the phase of secondary damage or improved axonal remyelination or a combination of both. A possible mechanism for neuroprotection is related to a buffering function of alginate hydrogel on the extracellular ions that are released after the injury and trigger excitotoxicity and apoptosis. This theory is supported by high sensitivity of alginate hydrogel to Ca^2+^ when incubated in cerebrospinal fluid–like media [21]. Increased remyelination might be related to the reduction of chondroitin sulphate proteoglycan (CSPG), which is an axonal growth inhibitory molecule, as it was already shown that CSPG was reduced upon the presence of alginate gel at the spinal cord contusion injury [44].

The development of a fibrotic scar is a further hallmark of lacerating SCI. A fibrous scar composed by a dense collagen IV meshwork creates a barrier for the regenerating axons [2] and is thus a therapeutic target.

**Fig 3:**
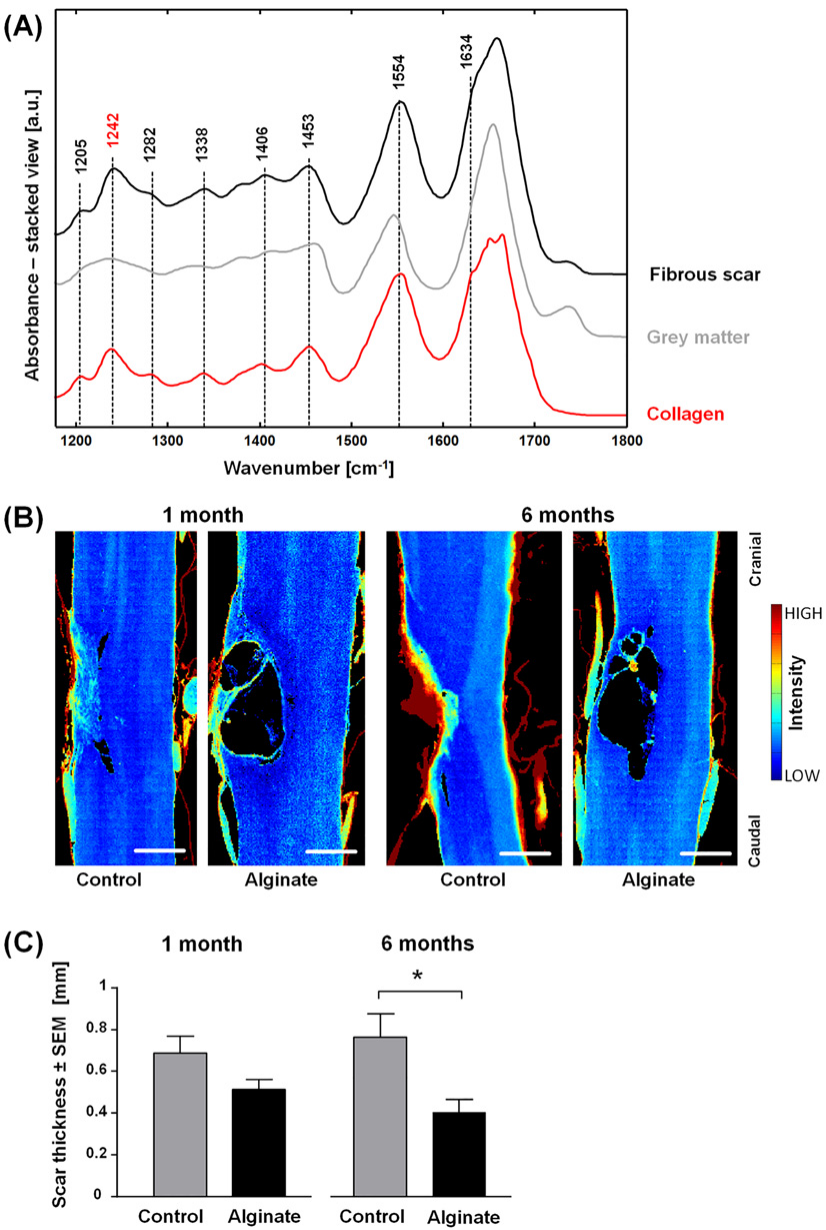
Analysis of the fibrotic lesion. (A) Representative IR spectra of the fibrous scar six months after injury, of grey matter and of reference collagen. (B) Spectroscopic images showing the intensity of the band 1242 cm^-1^ in pseudo color. They depict the distribution of collagen in control and alginate-implanted samples one and six months post-injury. Scale bar: 0.5 mm. (C) Fibrous scar thickness of control and alginate-implanted samples at one and six months post-injury, n = 5–7, two-tailed t-test, *: p < 0.05.

Fig 3A shows the IR spectra of the fibrous lesion and of gray matter. In the spectra of the fibrous scar, several bands were observed in the region of 1200–1450 cm^-1^ that were attributed to collagen, as it can be seen by comparison with the reference spectrum of pure collagen. In particular, the bands at 1205, 1242, 1282, and 1338 cm^-1^ are assigned to amide III, the band at 1406 cm^-1^ is assigned to δ[(CH_3_)] and the band at 1453 cm^-1^ to δ[(CH_2_)] and δ[(CH_3_)] [45,46]. Further spectral features observed in the spectra of the fibrotic lesion that are in common with collagen are the shoulder of the amide I band at 1634 cm^-1^ and a shift of the amide II band to 1554 cm^-1^. Therefore, collagen constitutes the main component of the fibrous scar. Minor contributions in the spectral region 1200–1500 cm^-1^ and at 1735 cm^-1^ arise from CNS nervous tissue. According to other studies [47], these spectral contributions origin most likely from the strong glial response after SCI that includes reactive gliosis and upregulation of chondroitin sulfate proteoglycans.

To investigate whether implantation of alginate affects scarring, the quantity of collagen that formed at the lesion site after SCI was analyzed. The intensity of the band at 1242 cm^-1^ was used to retrieve spectroscopic images that show the location and the extent of the scar: representative examples are shown in Fig 3B and the images of all samples are shown in S6 Fig. While the CNS nervous tissue scarcely contains collagen and is therefore characterized by very low intensities, variable amounts of collagen were localized at the injury site in all samples. In agreement with previous studies [2] the fibrous scar developed in the center of the lesion. Furthermore, meningeal tissue that contains high quantities of collagen is correctly detected at the border of the spinal cord.

The inspection of the spectroscopic images showed that the fibrous tissue tended to fill the lesion completely in the control samples, thus forming a dense collagen structure as shown exemplarily in the six-month control sample in Fig 3B. In contrast, alginate-implanted samples tended to have thinner layers of collagen mainly surrounding the cysts. The mean thickness of collagenous tissue was therefore quantified and was found to be significantly reduced in samples with alginate implant six months after injury compared to the untreated controls (Fig 3C, t-test, p = 0.0153). This indicates that the alginate hydrogel decreases the formation of the fibrous scar in the chronic stage of SCI.

Our results are in line with other studies showing that the introduction of alginate implants reduced the formation of collagen meshwork surrounding traumatic injury in CNS nervous tissue [20,48]. In case of lacerating injury, the damage of the dura allows the infiltration of meningeal fibroblasts, which further contribute to the scarring by accumulation of connective tissue [2]. An underlying cause for the reduced fibrotic scarring might be the relatively poor adhesion of fibroblasts on pure alginate hydrogel [49].

The soft Ca^+2^-alginate hydrogel used in these experiments is characterized by gelation of 5% of the gelation sites and shear storage modulus G′ = 0.195 kPa (S7 Fig). This constitutes a substantial difference compared to alginates investigated in vivo so far and was considered crucial for the ability of the material to support neural adhesion and the abundant neurite outgrowth that was observed in vitro [29]. As multiple polysaccharide sequences remain uninvolved in the cross-linking reaction, they can potentially interact with cell surface molecules [29]. Moreover, even though this type of alginate hydrogel was found to maintain its mass and volume after immersion in saline solution [30], the infiltration of exogenous sodium ions might lead to leakage of calcium ions and finally to hydrogel dissolution [50]. Therefore the persistence of implants and ion exchange were investigated.

**Fig 4:**
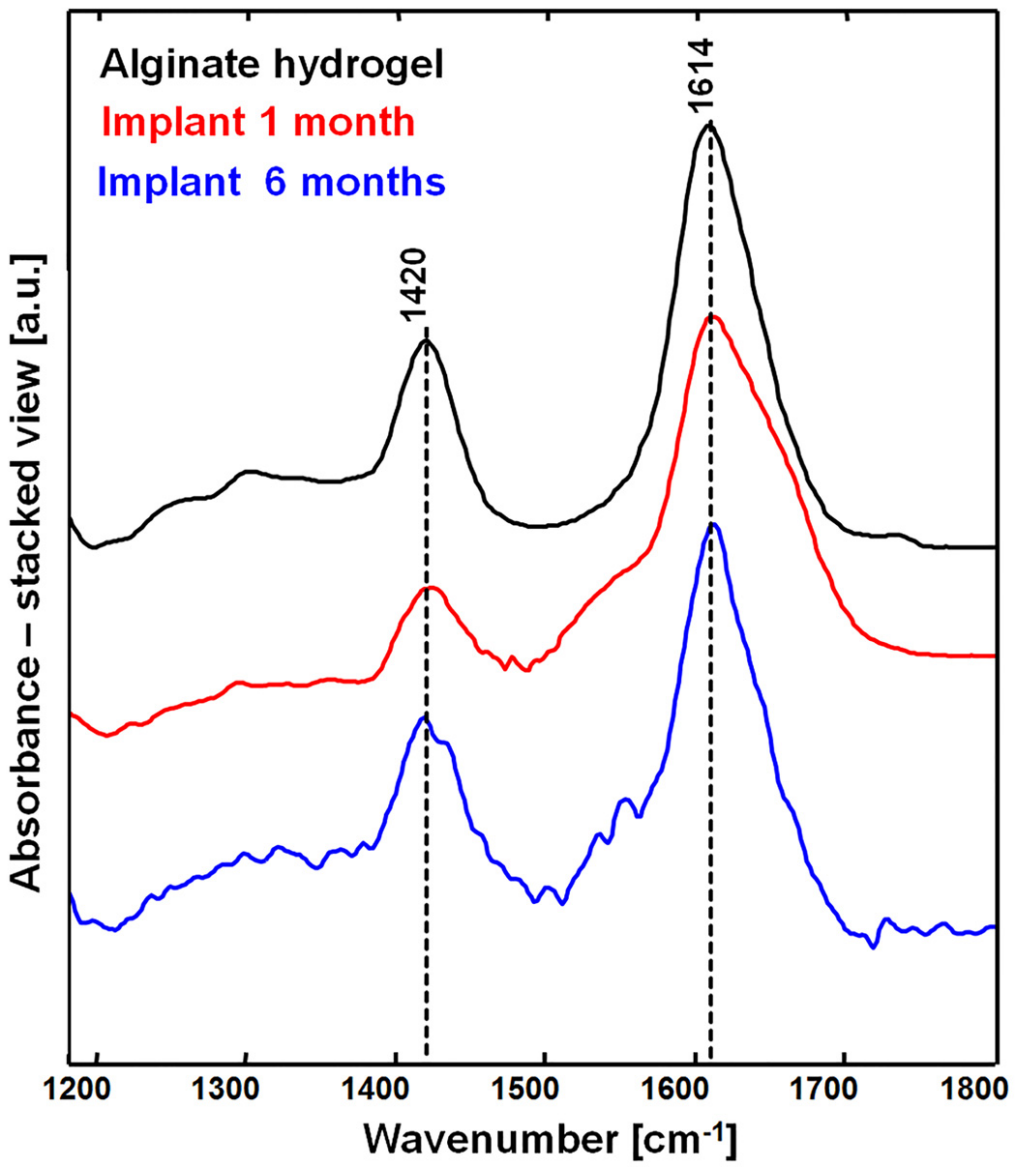
FT-IR spectroscopy of the alginate hydrogel implants. IR spectrum of pure alginate hydrogel before its implantation (black) compared with the spectrum of alginate hydrogel in spinal cord tissue at one (red) and six months after injury (blue).

The IR spectrum of native (i.e. before implantation) alginate hydrogel is shown in Fig 4 (black spectrum). It exhibits prominent bands at 1614 and 1420 cm^-1^ that are assigned to carboxylate salt ion antisymmetric and symmetric stretching [51]. The chemical structure of Ca^2+^-alginate hydrogel is shown in S8 Fig for reference.

Residues of the implants were identified in the spinal cord samples one month (6/7) and six months (5/7) after injury. The IR spectra of the implanted alginate were strongly overlapped with spectral contributions of sucrose. Therefore, the difference spectra of implanted alginate and reference sucrose were used for further analysis and are shown in Fig 4 (one month after injury: red; six months after injury: blue).

The overall spectral pattern of implanted and pure alginate is similar. In particular, the positions of bands at 1614 cm^-1^ and 1420 cm^-1^ were consistent. As the position of these bands is relevant for the discrimination of alginate structures from their chemical derivatives [52], a lack of shifts indicates that no exchange of crosslinking ions took place in the hydrogel after implantation. In the spectrum of implanted alginate, shoulders at approximately 1550 and 1650 cm^-1^ were observed. They are consistent with amide II and I respectively, indicating a mixing of alginate with proteins. No spectral shifts between the spectra were observed among samples of one and six months implanted alginate hydrogels, confirming that the implants retain their chemical structure over the time. Nevertheless, variations of the mechanical properties of implants cannot be excluded.

The results agree with the slow degradation rate of calcium alginate hydrogels, which take several months to lose stability under physiological conditions [53]. As there are no endogenous enzymes in rodents which digest alginate hydrogel in spinal cord, its degradation is caused by gradual diffusion of calcium ions resulting in unlinking and dissolution of alginate polymer chains [50]. The damage of CNS nervous tissue causes elevated levels of ions in the extracellular matrix [54], which enhances the rate of diffusion of Ca^2+^ cross–linkers within the hydrogel. Depending on the intensity of extracellular ions influx, the hydrogel degradation rate might vary among the samples and this could explain why the hydrogel was not found in all samples.

In order to gain information on the distribution of alginate inside the tissue, RGB spectroscopic images were generated using the intensity of specific bands that show the distribution of the therapeutic implant, of the nervous tissue and of the fibrous scar. The band at 1420 cm^-1^ is highly specific for alginate (compare Fig 4) and was selected to visualize the alginate implant in the spinal cord samples. Nervous tissue was identified by the amide I band at 1653 cm^-1^ (compare Fig 1), and the scar was visualized using the band at 1242 cm^-1^ that is assigned to collagen (compare Fig 3).

**Fig 5:**
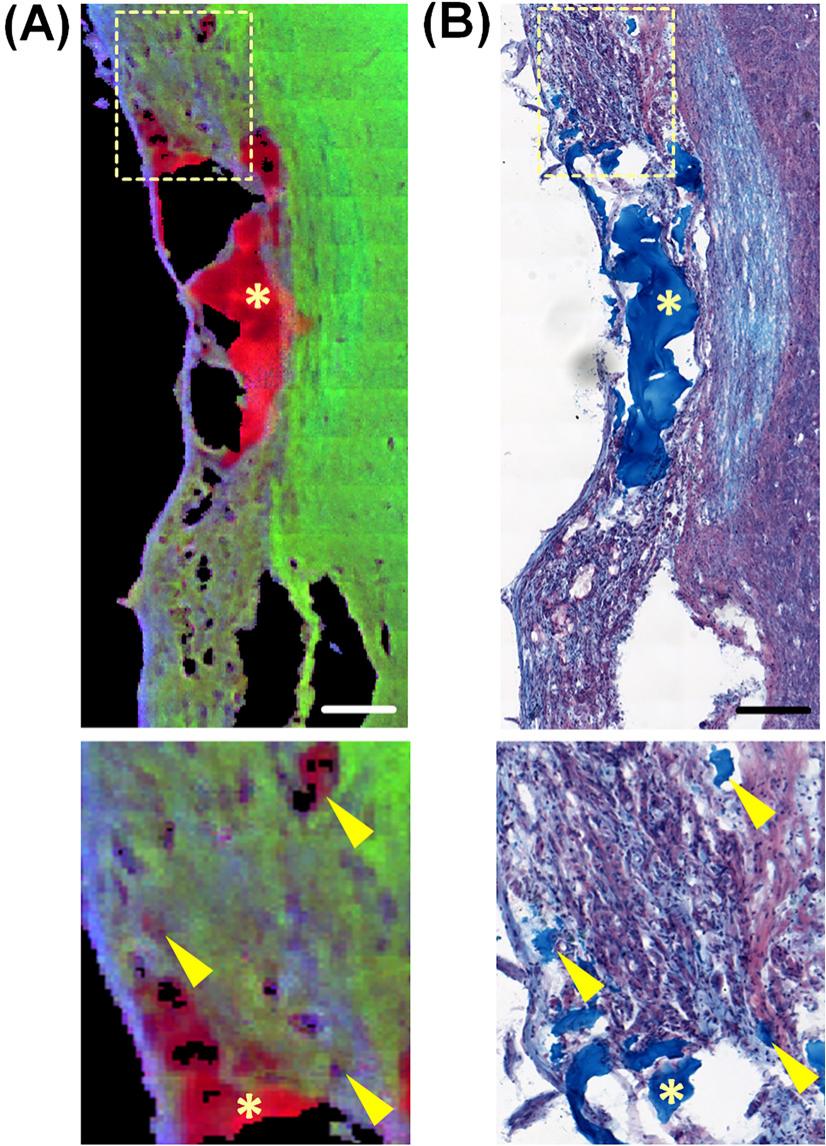
Distribution of alginate within the tissue six months after injury. (A) spectroscopic RGB image, generated by combining the intensity of bands at 1420 cm^-1^ (alginate hydrogel–red), 1653 cm^-1^ (nervous tissue–green) and 1242 cm^-1^ (collagen—blue). (B) Alcian blue staining of a consecutive section. Scale bars: 100 μm. The boxes in A and B indicate the area of magnification. The arrowheads in the magnifications indicate small inclusions of alginate hydrogel into the tissue that colocalize in the spectroscopic image and in the staining. The asterisks indicate alginate inside large cysts.

An example of a RGB spectroscopic image of an spinal cord six months after injury that received an alginate implant is shown in Fig 5A (red: alginate hydrogel, green: nervous tissue, blue: fibrous tissue). Small cysts of variable dimension containing alginate hydrogel were observed within the lesion and correspond with the reference alcian blue staining for alginate (Fig 5B, indicated by arrowheads). This indicates a growth of tissue through the alginate hydrogel. Moreover residues of alginate without nervous tissue ingrowth fill the larger cysts (Fig 5, indicated by asterisks), indicating that the nervous tissue was not able to completely bridge across the alginate-filled lesions. It is well known that large cysts constitute a physical barrier to the regenerating axons [55]. On the other side, we showed that the collagenous scar surrounding these cysts in the treated animals is generally thin, while an extended collagenous scar–which is also a barrier to regenerating axons–tends to fill the lesion site of untreated animals. Therefore the presence of large alginate-filled cysts is not indicating *per se* a worse outcome of the treated animals.

## Conclusions

IR spectroscopic imaging of cryosections of SCI in rat model provides biochemically detailed insights into pathological CNS tissue alterations and enables to assess the therapeutic effect of non-functionalized low-gelation soft Ca^2+^-alginate hydrogel implants. Instead of performing multiple immunohistochemical stainings on consecutive sections, this technique enables to address the different aspects of SCI in one dataset. We showed that the implants persist in the living CNS nervous tissue environment, as they did not decompose up to six months after implantation. No negative side effects of the implant were observed. Furthermore, we found that the overall tissue architecture and morphology of alginate-implanted samples appeared more preserved, presumably thanks to less tissue shearing and compression. These findings suggest that alginate hydrogel serves also as a mechanical support to preserve the gross spinal cord morphological structure and may help to limit secondary damage of nervous tissue. The number of animals investigated allowed the quantitative assessment of tissue damage and revealed significant differences of the tissue’s molecular composition between the control and alginate-treated group six months after SCI.

The results demonstrate that FT-IR spectroscopy is a valid tool to complement standard biological techniques for the assessment of SCI treatment. FT-IR imaging enabled to show that non-functionalized low-gelation soft Ca^2+^-alginate hydrogel has an enhanced long-term stability *in vivo* and suggests that the implant improves the outcome by limiting demyelination of CNS nervous tissue and by reducing scarring at the chronic stage of SCI.

## Supporting Information

S1 Fig Workflow of implantation procedure. After cross-linking, the alginate hydrogel in a 6 cm dish is overlaid with a 10 ml of 4 mM CaCl2 and 150 mM NaCl solution; within 1 h it is removed from the dish and manually cut in blocks of 2 mm x 2 mm x 1.5 mm using a scalpel. The spinal cord is exposed by laminectomy and a 2 mm long and 1.5 mm deep hemisection is produced; the hydrogel block is inserted in the hemisection with the help of a surgical micro-spoon.
(TIF)

S2 Fig Assessment of scar extension. The intensities of the spectral band at 1242 cm^-1^ were used to retrieve the distribution of collagen in the cryosection (here displayed as gray scale image). The intensity profile (shown in red) was calculated along a line crossing the scar center (dotted yellow line). Pixels characterized by an intensity values above the threshold of 0.025 (corresponding to the max. background value of the nervous tissue among all samples) were assigned to fibrous tissue and used to evaluate the thickness of the scar. The dura left at the sample borders was excluded from the calculation.
(TIF)

S3 Fig H&E stained sections of SCI in rat models with and without alginate hydrogel implant at one and six months after injury. (TIF)

S4 Fig Representative IR spectra of white and grey matter, and IR spectrum of sucrose. In the spectral region comprised between 900 and 1180 cm^-1^ the contribution of nervous tissue is overwhelmed by the contribution of sucrose.
(TIF)

S5 Fig Analysis of the contralateral nervous tissue. IR spectroscopic images of SCI in rat models with and without alginate hydrogel implant at one and six months after injury, obtained plotting the intensity of the lipid-related band at 1735 cm^-1^.
(TIF)

S6 Fig Analysis of the fibrotic lesion. IR spectroscopic images of SCI in rat models with and without alginate hydrogel implant at one and six months after injury, obtained plotting the intensity of the collagen-related band at 1242 cm^-1^.
(TIF)

S7 Fig Storage modulus vs. deformation of 4% alginate crosslinked in a 4 mM CaCl2 solution. (TIF)

S8 Fig Chemical structure of Ca^2+^-alginate hydrogel. Alginate is a negatively charged linear polysaccharide, composed of covalently linked D-mannuronic acid and L-guluronic acid monomers. It forms hydrogels by physical interaction of polymer chains via ionic cross-linking by multivalent cations at the sites of guluronic acid monomers sequences between different chains.
(TIF)
