# Correction: Biochemical Monitoring of Spinal Cord Injury by FT-IR Spectroscopy—Effects of Therapeutic Alginate Implant in Rat Models

**DOI:** 10.1371/journal.pone.0150237

**Published:** 2016-02-22

**Authors:** Sandra Tamosaityte, Roberta Galli, Ortrud Uckermann, Kerim H. Sitoci-Ficici, Robert Later, Rudolf Beiermeister, Falko Doberenz, Michael Gelinsky, Elke Leipnitz, Gabriele Schackert, Edmund Koch, Valdas Sablinskas, Gerald Steiner, Matthias Kirsch

The terms “cranial” and “caudal” are incorrectly switched in the article text and in Figs 1, 2, 3, and Supporting S3, S5 and S6 Figs. Please view the corrected figures here.

**Fig 1:**
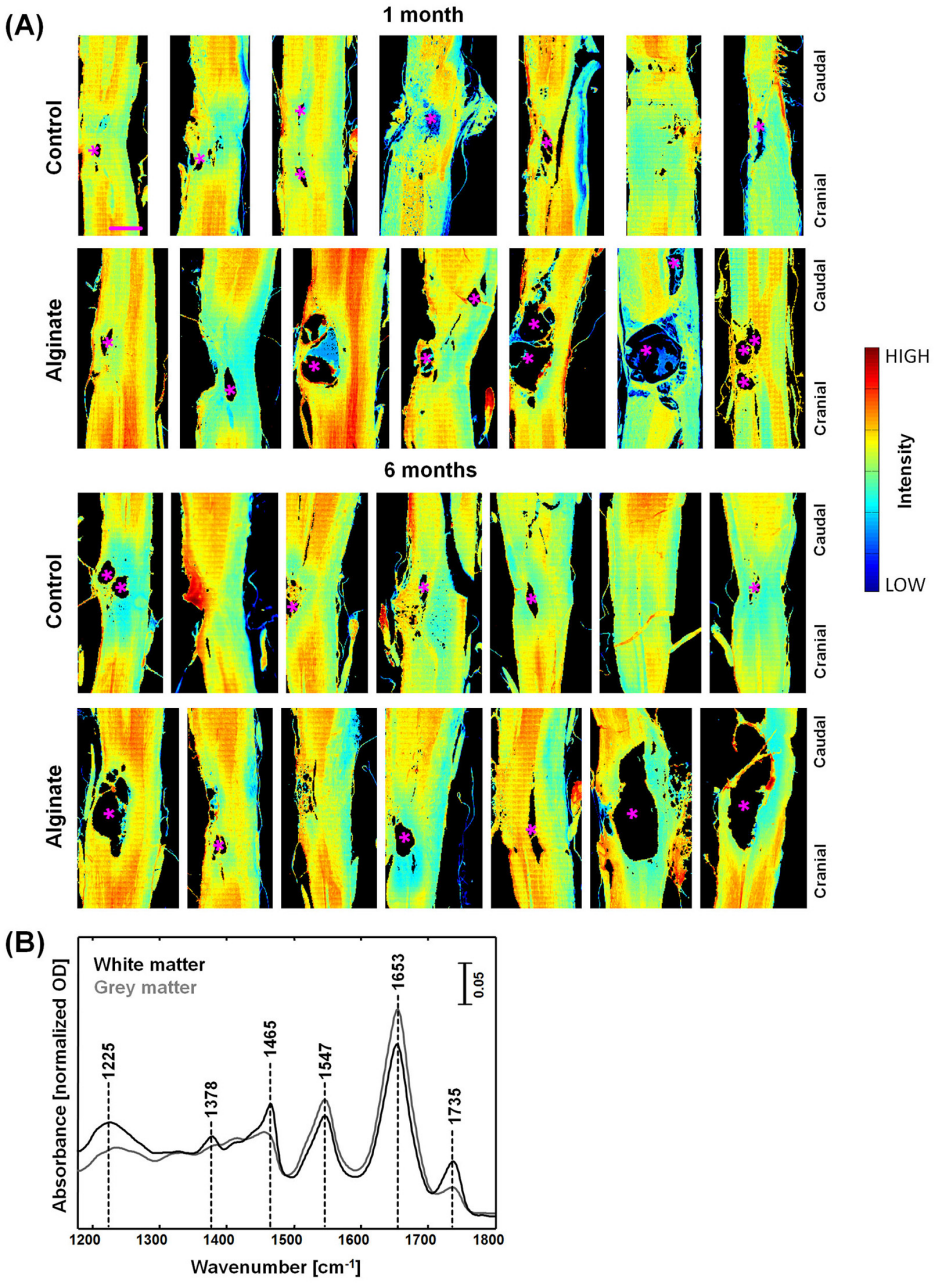
IR spectroscopic imaging of SCI in rat models with and without alginate hydrogel implant at one and six months after injury. (A) Spectroscopic images of longitudinal cryosections of the investigated samples, showing the intensity of the amide I band at 1653 cm^-1^; asterisks (*) indicate large cysts. Scale bar: 1 mm. (B) Representative IR spectra of white and grey matter.

**Fig 2:**
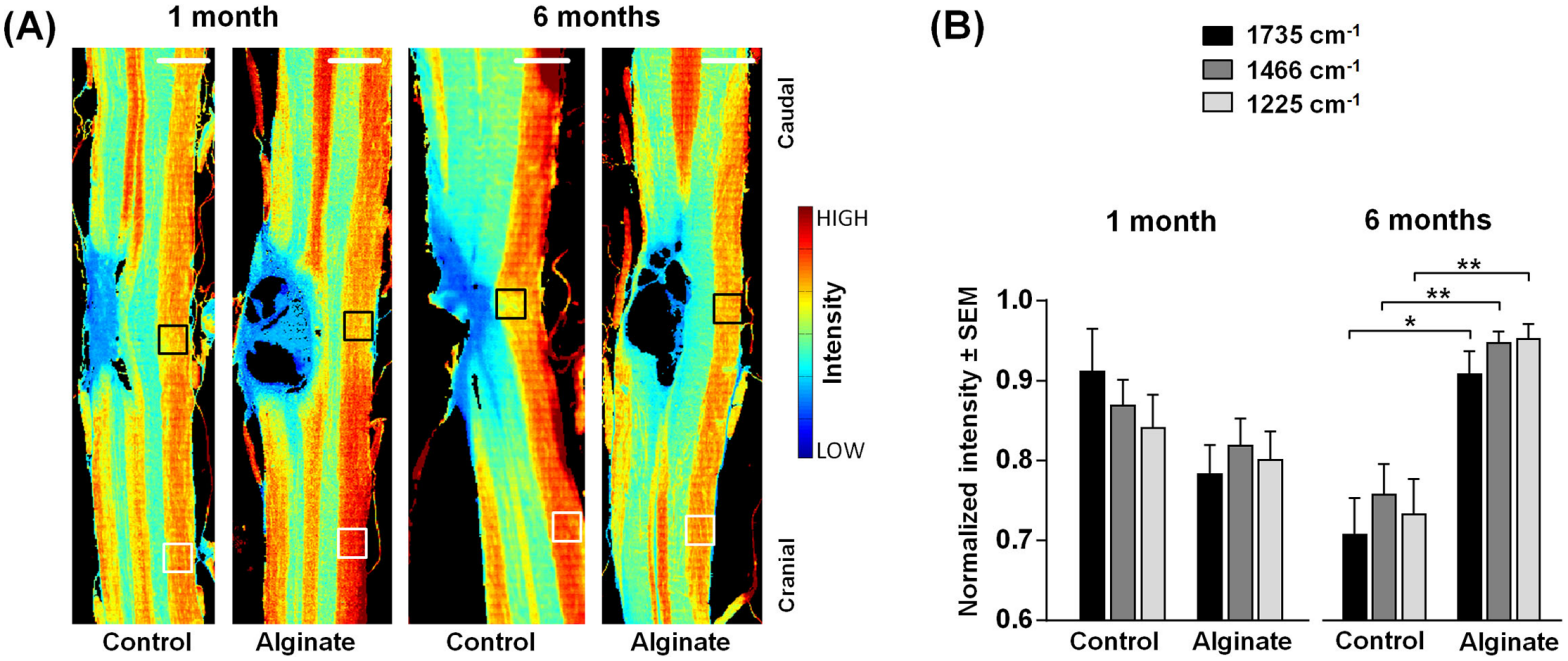
Analysis of the contralateral nervous tissue. (A) Spectroscopic images showing the intensity of the band at 1735 cm^-1^ (ν_s_(C = O)). They depict the distribution of lipids in control and alginate-implanted samples one and six months post-injury. Scale bar: 1 mm. (B) Intensities of the bands at 1735 cm^-1^, 1466 cm^-1^ (δ[(CH_2_)], also showing the distribution of lipids) and 1225 cm^-1^ (ν_as_(PO_2_^−^), showing the distribution of phospholipids) in the white matter contralateral to the lesion indicated by the black boxes in panel A normalized for each samples to the intensity in the region indicated by white boxes in panel A. For each group n = 5–6. Two-tailed t-test, *: p < 0.05, **: p < 0.01.

**Fig 3:**
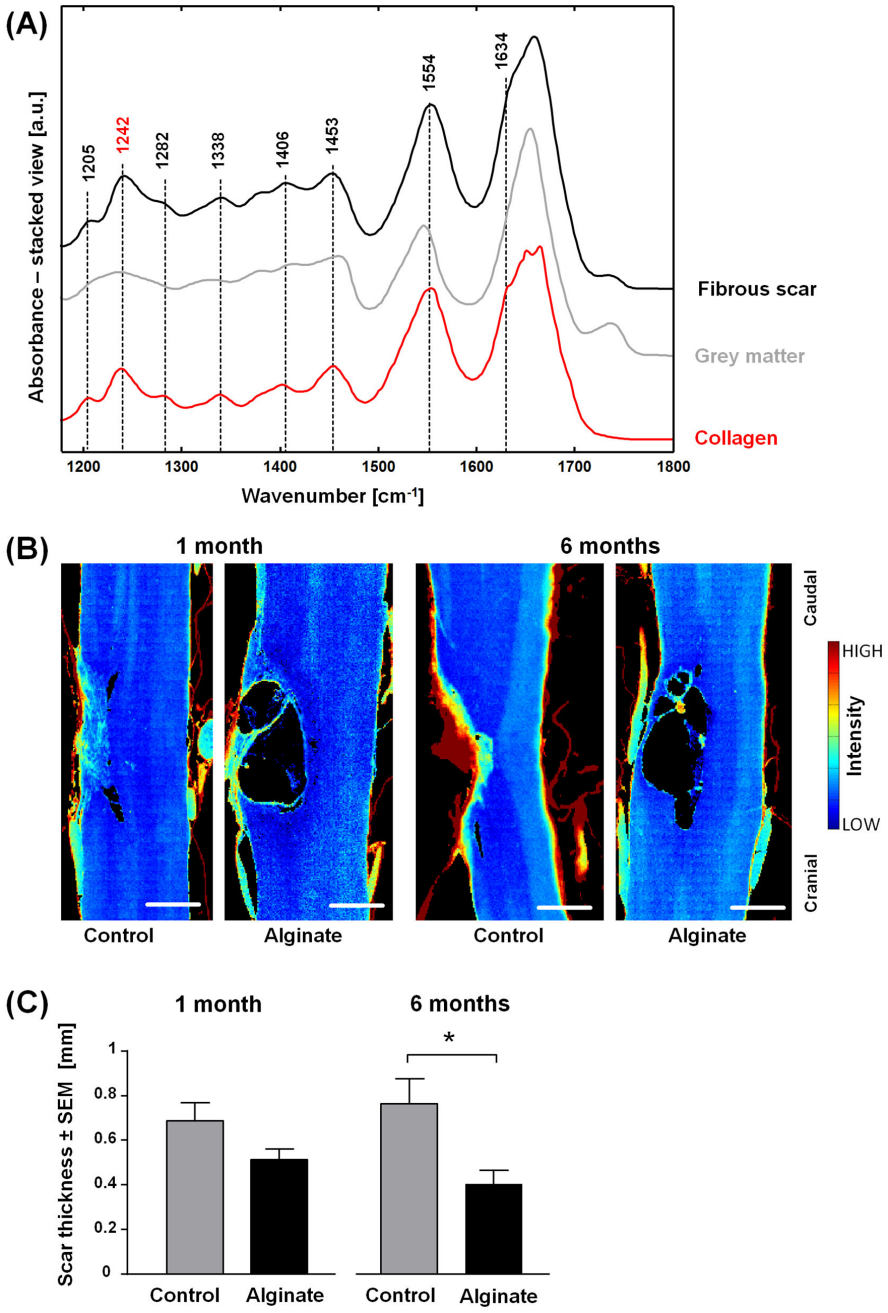
Analysis of the fibrotic lesion. (A) Representative IR spectra of the fibrous scar six months after injury, of grey matter and of reference collagen. (B) Spectroscopic images showing the intensity of the band 1242 cm^-1^ in pseudo color. They depict the distribution of collagen in control and alginate-implanted samples one and six months post-injury. Scale bar: 0.5 mm. (C) Fibrous scar thickness of control and alginate-implanted samples at one and six months post-injury, n = 5–7, two-tailed t-test, *: p < 0.05.

In the “Data processing and analysis” section of the Materials and Methods, the third sentence of the third paragraph should read: Mean band intensities were normalized to the intensities calculated the same white matter tract cranial to the lesion (4.5 mm away from the hemisection center).

## Supporting Information

S3 Fig H&E stained sections of SCI in rat models with and without alginate hydrogel implant at one and six months after injury. (JPG)

S5 Fig Analysis of the contralateral nervous tissue. IR spectroscopic images of SCI in rat models with and without alginate hydrogel implant at one and six months after injury, obtained plotting the intensity of the lipid-related band at 1735 cm^-1^.
(JPG)

S6 Fig Analysis of the fibrotic lesion. IR spectroscopic images of SCI in rat models with and without alginate hydrogel implant at one and six months after injury, obtained plotting the intensity of the collagen-related band at 1242 cm^-1^.
(JPG)
