# Supplementary figures and images for: Biochemical Monitoring of Spinal Cord Injury by FT-IR Spectroscopy—Effects of Therapeutic Alginate Implant in Rat Models
Source: PLoS One. 2015 Nov 11;10(11):e0142660. doi: 10.1371/journal.pone.0142660 (PMC4641584; doi:10.1371/journal.pone.0142660)

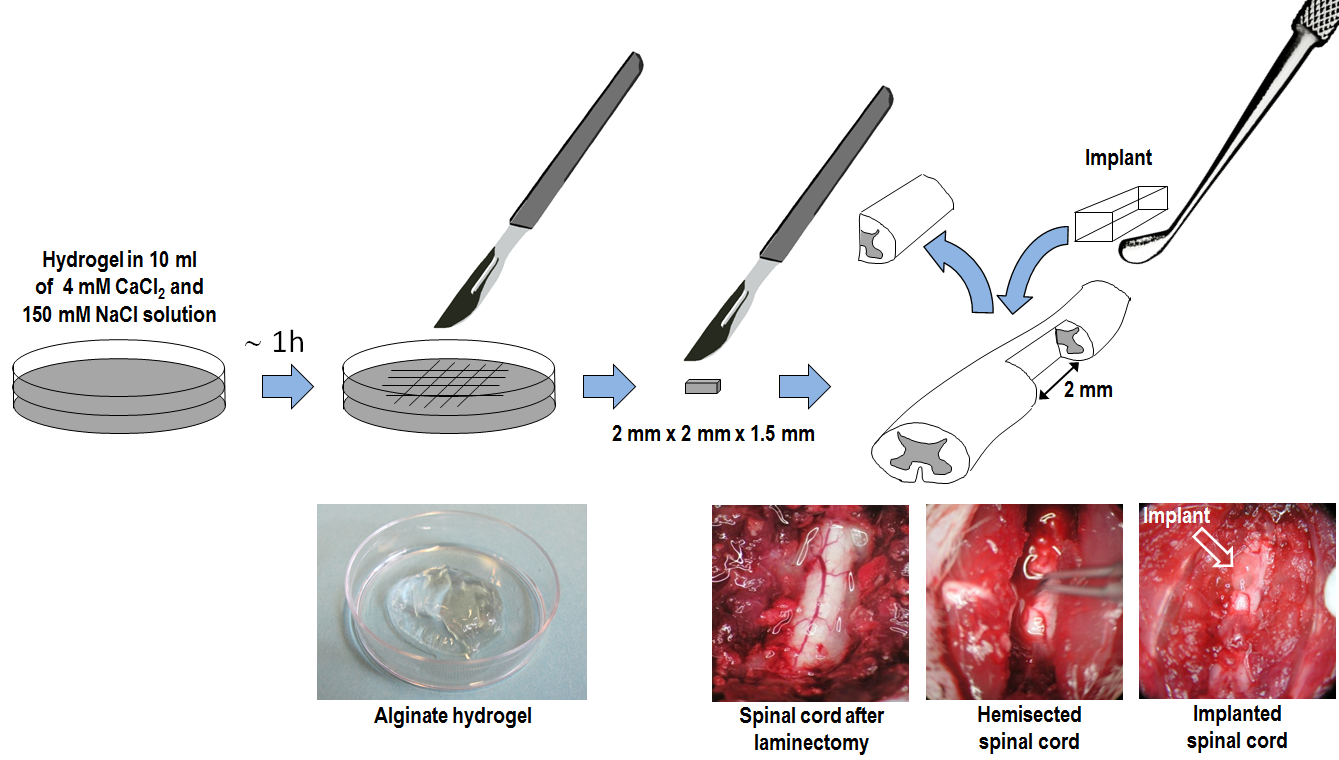

Supplement: S1 Fig — After cross-linking, the alginate hydrogel in a 6 cm dish is overlaid with a 10 ml of 4 mM CaCl2 and 150 mM NaCl solution; within 1 h it is removed from the dish and manually cut in blocks of 2 mm x 2 mm x 1.5 mm using a scalpel. The spinal cord is exposed by laminectomy and a 2 mm long and 1.5 mm deep hemisection is produced; the hydrogel block is inserted in the hemisection with the help of a surgical micro-spoon. (TIF) [file pone.0142660.s001.tif]

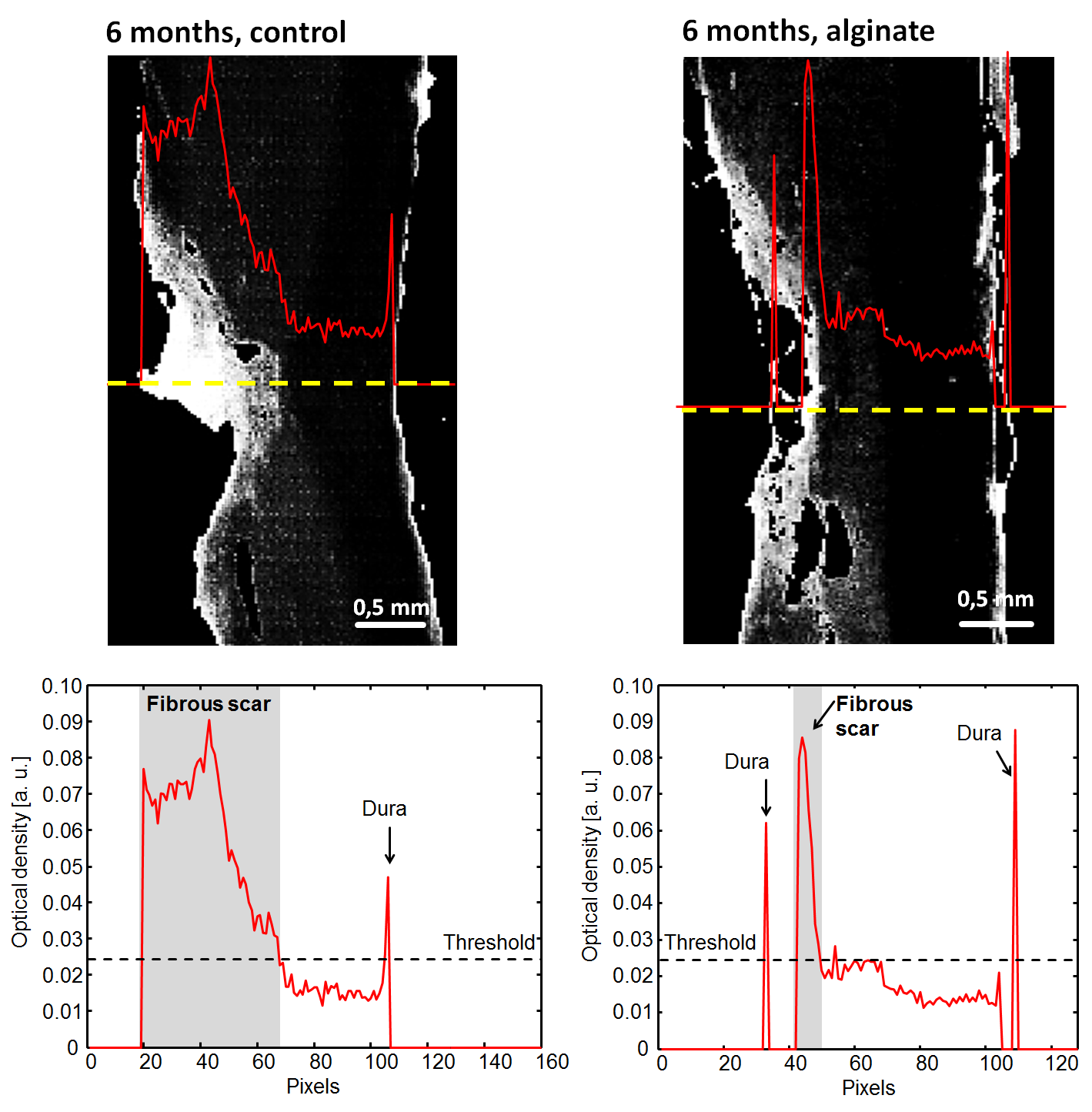

Supplement: S2 Fig — The intensities of the spectral band at 1242 cm-1 were used to retrieve the distribution of collagen in the cryosection (here displayed as gray scale image). The intensity profile (shown in red) was calculated along a line crossing the scar center (dotted yellow line). Pixels characterized by an intensity values above the threshold of 0.025 (corresponding to the max. background value of the nervous tissue among all samples) were assigned to fibrous tissue and used to evaluate the thickness of the scar. The dura left at the sample borders was excluded from the calculation. (TIF) [file pone.0142660.s002.tif]

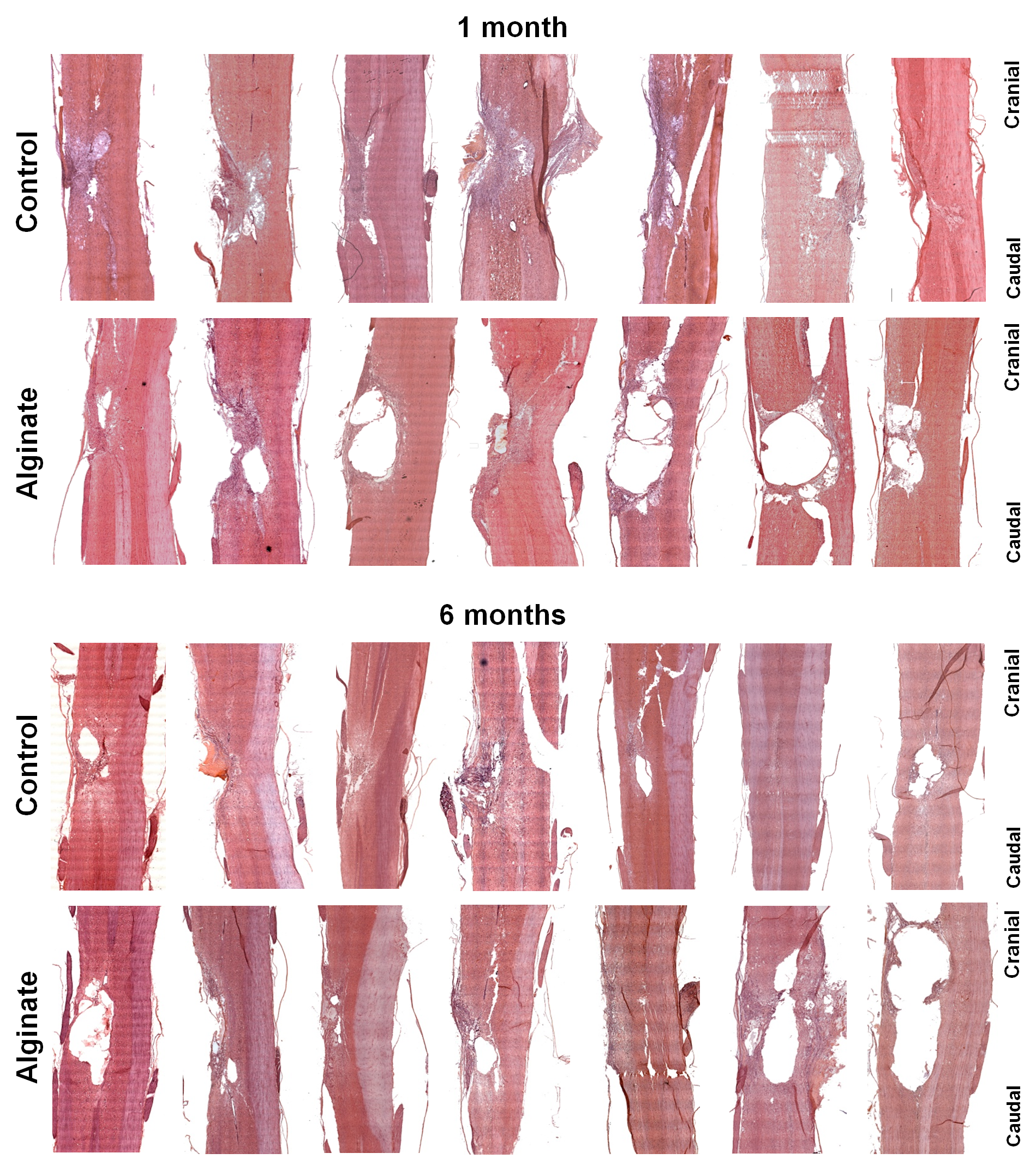

Supplement: S3 Fig — (TIF) [file pone.0142660.s003.tif]

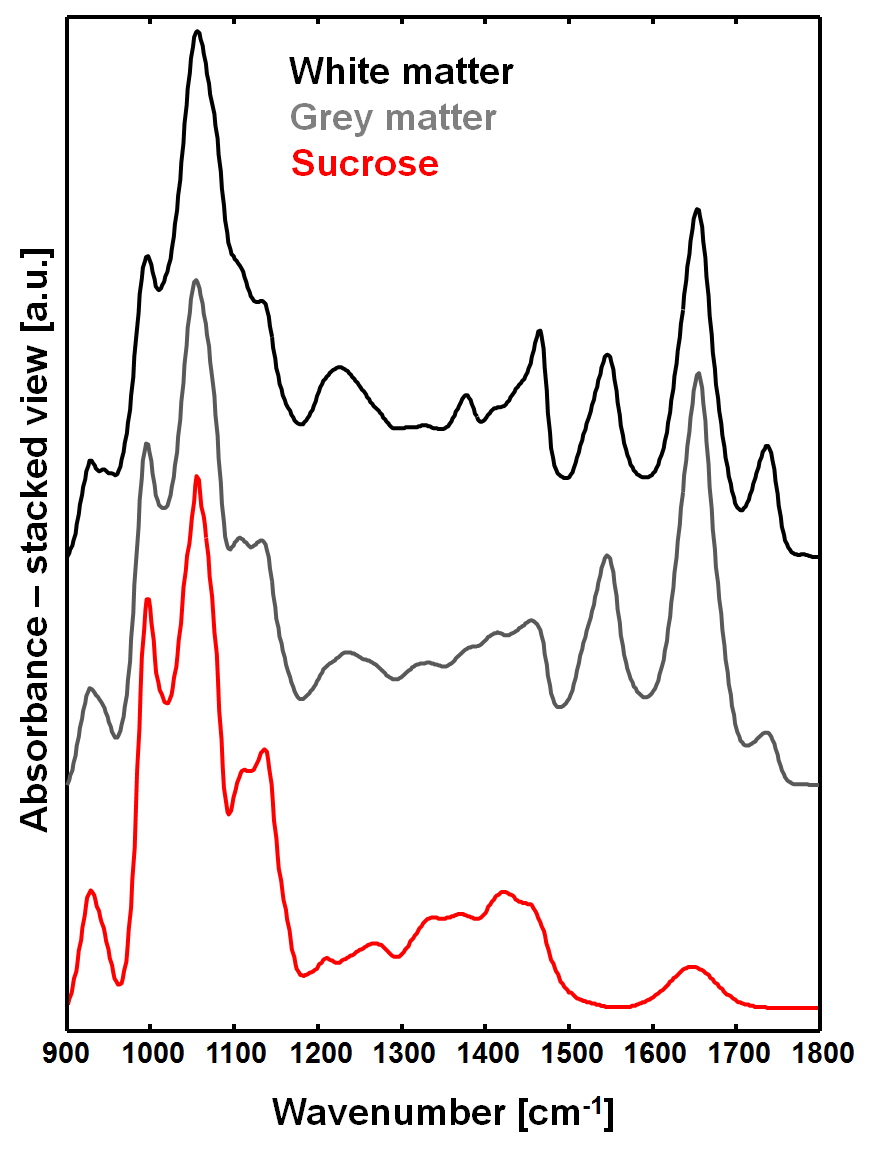

Supplement: S4 Fig — In the spectral region comprised between 900 and 1180 cm-1 the contribution of nervous tissue is overwhelmed by the contribution of sucrose. (TIF) [file pone.0142660.s004.tif]

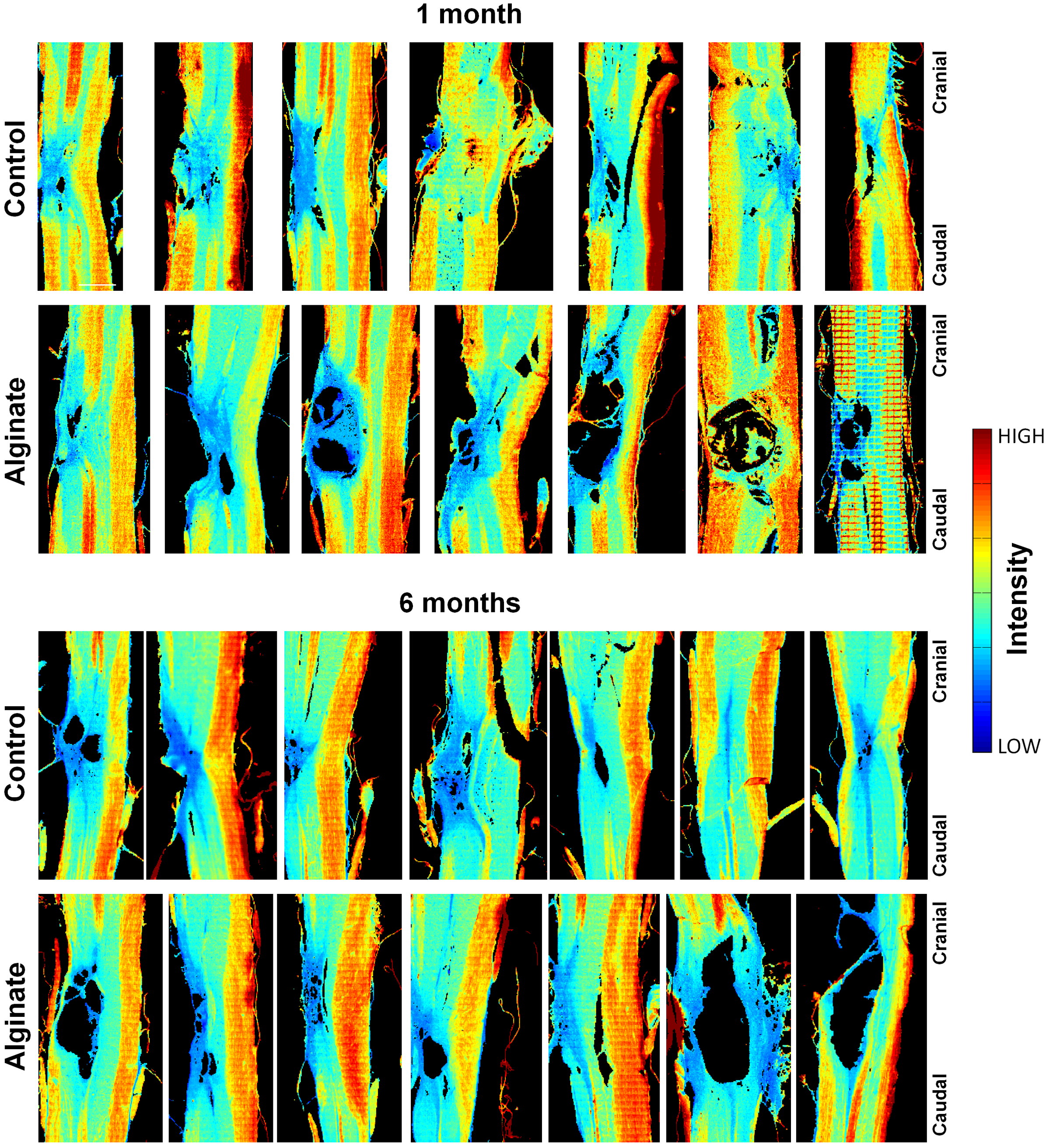

Supplement: S5 Fig — IR spectroscopic images of SCI in rat models with and without alginate hydrogel implant at one and six months after injury, obtained plotting the intensity of the lipid-related band at 1735 cm-1. (TIF) [file pone.0142660.s005.tif]

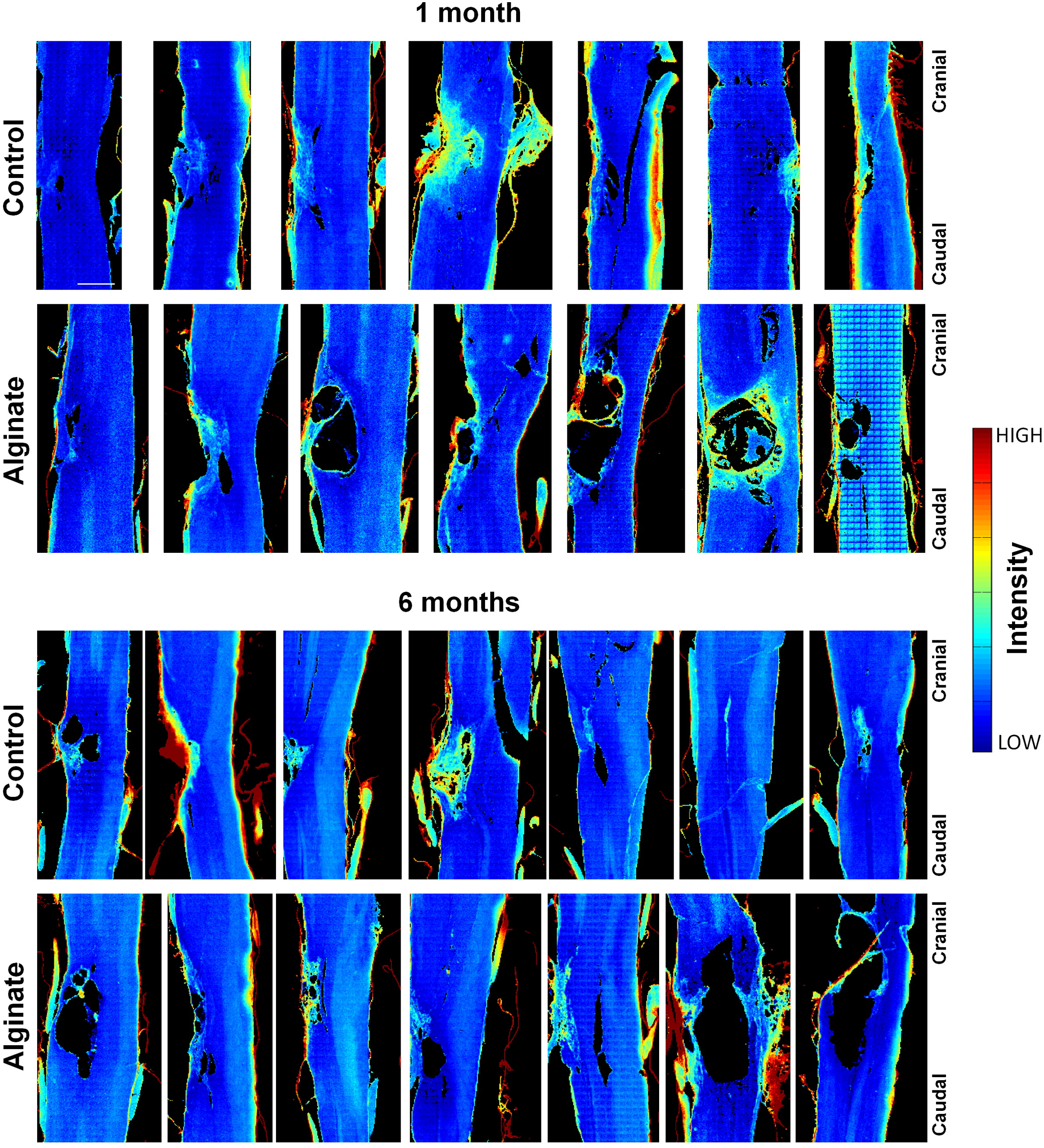

Supplement: S6 Fig — IR spectroscopic images of SCI in rat models with and without alginate hydrogel implant at one and six months after injury, obtained plotting the intensity of the collagen-related band at 1242 cm-1. (TIF) [file pone.0142660.s006.tif]

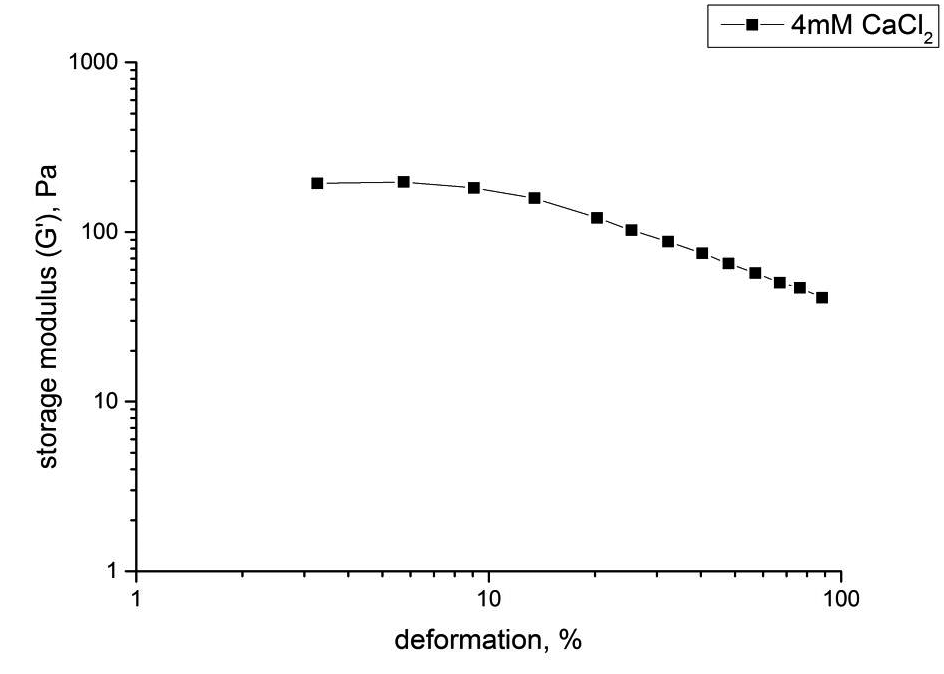

Supplement: S7 Fig — (TIF) [file pone.0142660.s007.tif]

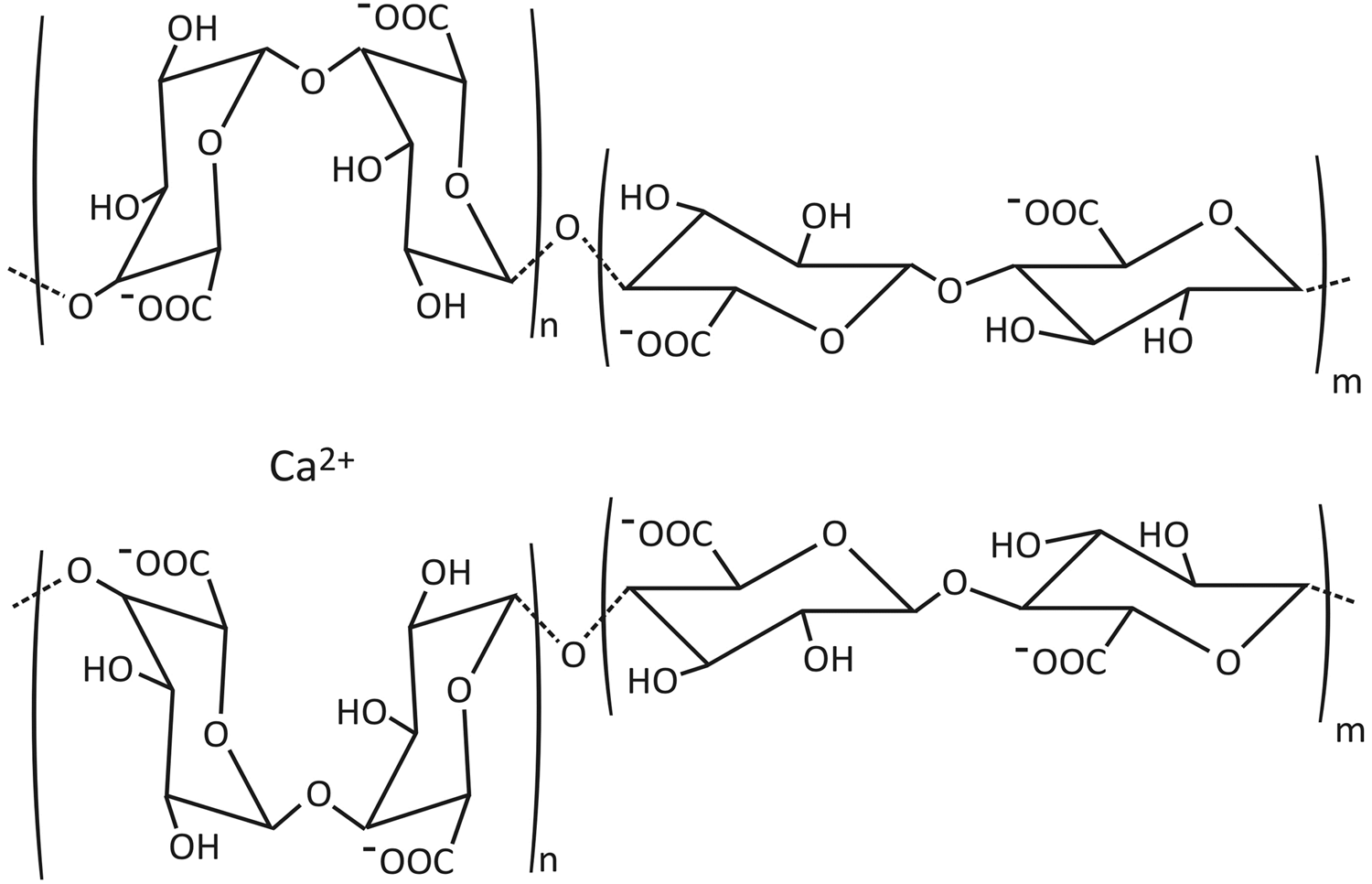

Supplement: S8 Fig — Alginate is a negatively charged linear polysaccharide, composed of covalently linked D-mannuronic acid and L-guluronic acid monomers. It forms hydrogels by physical interaction of polymer chains via ionic cross-linking by multivalent cations at the sites of guluronic acid monomers sequences between different chains. (TIF) [file pone.0142660.s008.tif]
