# Supplementary figures and images for: Correction: Biochemical Monitoring of Spinal Cord Injury by FT-IR Spectroscopy—Effects of Therapeutic Alginate Implant in Rat Models
Source: PLoS One. 2016 Feb 22;11(2):e0150237. doi: 10.1371/journal.pone.0150237 (PMC4762664; doi:10.1371/journal.pone.0150237)

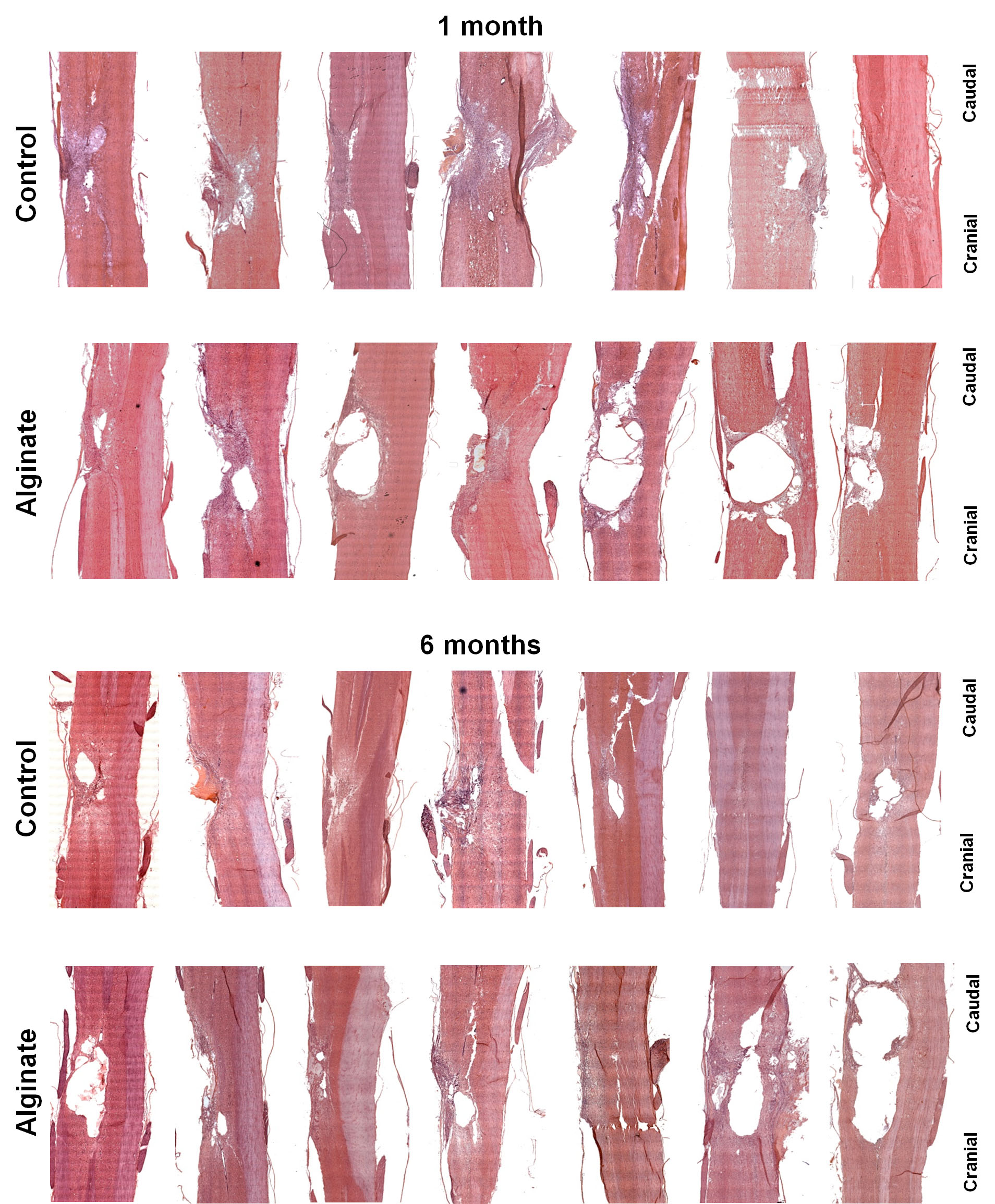

Supplement: S3 Fig — (JPG) [file pone.0150237.s001.jpg]

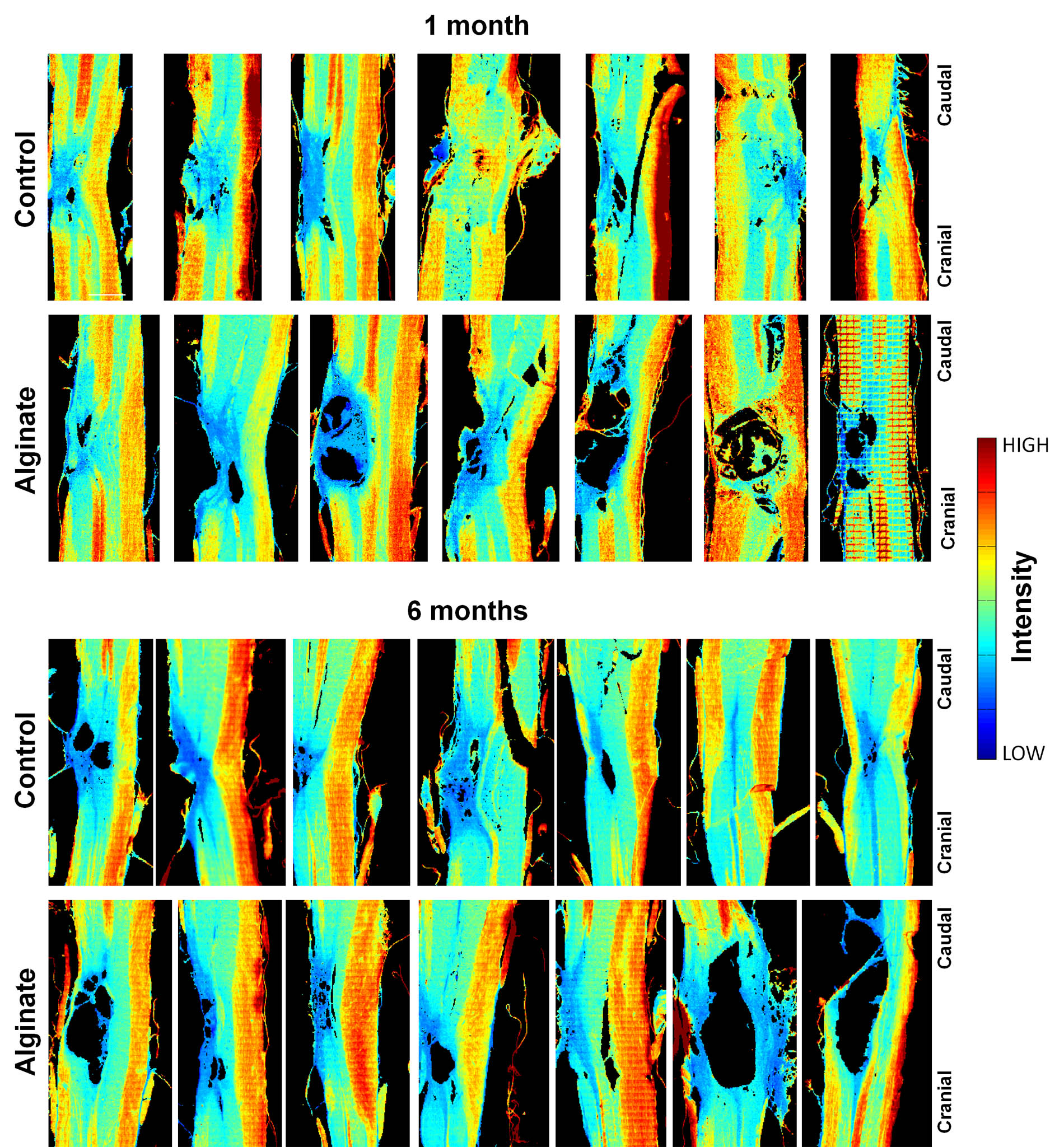

Supplement: S5 Fig — IR spectroscopic images of SCI in rat models with and without alginate hydrogel implant at one and six months after injury, obtained plotting the intensity of the lipid-related band at 1735 cm-1. (JPG) [file pone.0150237.s002.jpg]

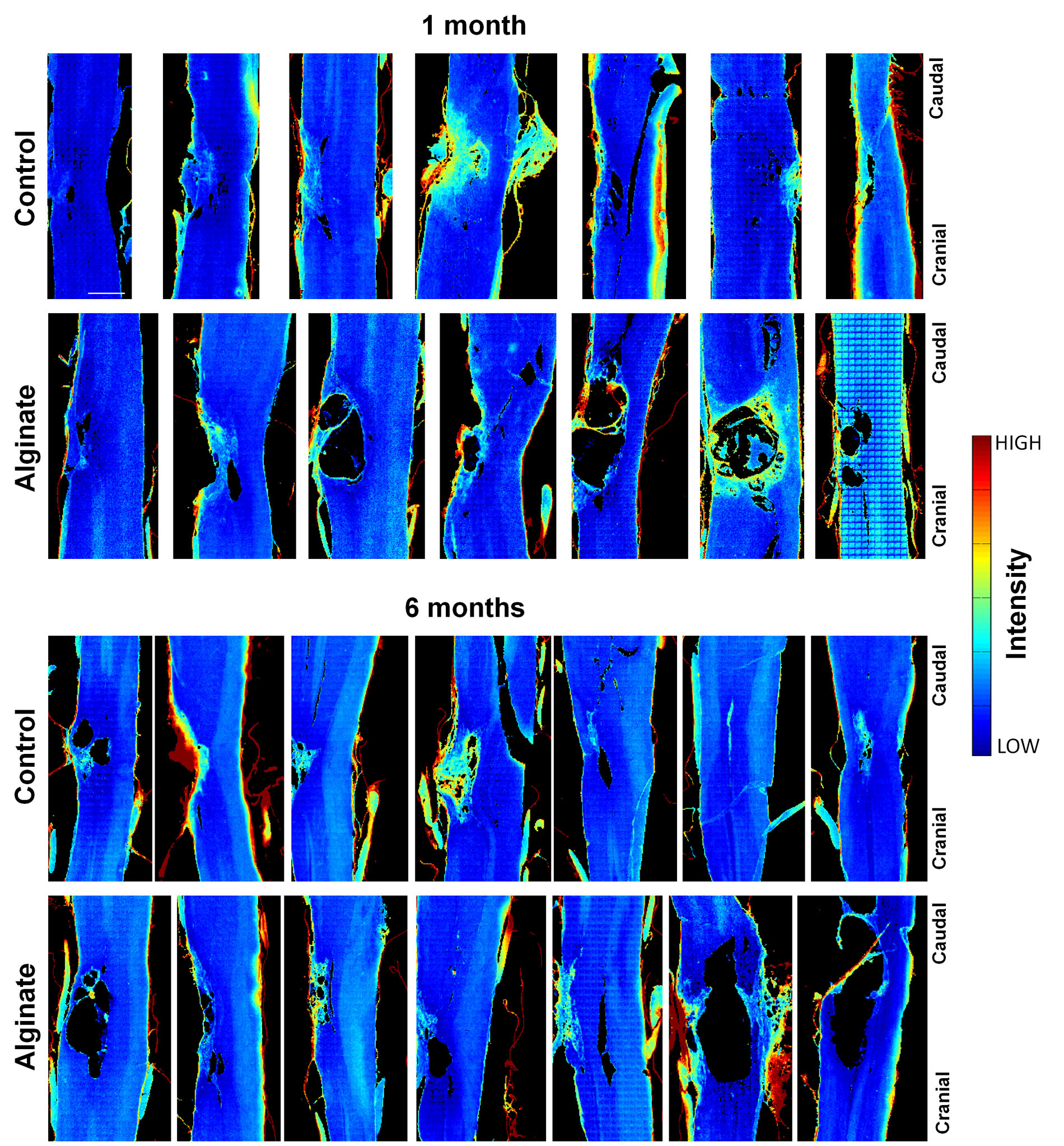

Supplement: S6 Fig — IR spectroscopic images of SCI in rat models with and without alginate hydrogel implant at one and six months after injury, obtained plotting the intensity of the collagen-related band at 1242 cm-1. (JPG) [file pone.0150237.s003.jpg]
